# The effect of different types of *in ovo* selenium injection on the immunity, villi surface area, and growth performance of local chickens

**DOI:** 10.14202/vetworld.2021.1109-1115

**Published:** 2021-05-07

**Authors:** Rantan Krisnan, Yuli Retnani, Budi Tangendjaja, Rita Mutia, Anuraga Jayanegara, Elizabeth Wina

**Affiliations:** 1 Study Program of Nutrition and Feed Science, Graduate School of IPB University, Bogor 16680, Indonesia; 2 Indonesian Research Institute for Animal Production, Bogor 16002, Indonesia; 3 Department of Nutrition and Feed Technology, Faculty of Animal Science, IPB University, Bogor 16680, Indonesia

**Keywords:** immunity, *in ovo*, local chicken, performance, selenium, villi

## Abstract

**Background and Aim:**

The presence of free radicals may lower chicken’s performance. Thus, the antioxidant defense is needed and can be made through a nutritional approach such as selenium supplementation before hatches. This study aimed to investigate the type of selenium that, as an *in ovo* feeding (IOF) material, can provide the most enhancement of immunity, villi surface area, and early growth performance of local chickens.

**Materials and Methods:**

This study, with a completely randomized design, used 480 fertile Kampung Unggul Balitbangtan (KUB, a selected local breed) chicken eggs, with 120 eggs per treatment for four treatments. The four treatments of IOF material included the treatment with organic selenium yeast (SY), organic hydroxy-selenomethionine (HSM), inorganic sodium selenite (SS), and uninjected selenium (control). A solution containing 0.15 ppm of different selenium was injected into the egg amnion after 18 days of incubation. Once hatched, the chicks were placed in three individual cages for each treatment (capacity of eight birds per cage). The parameters observed were the villi surface area, antibody titer, the number of total and differentiated leucocytes, glutathione peroxidase (GSH-Px) activity levels, and growth and feed efficiency of the early growth performance.

**Results:**

All the *in ovo* selenium feeding, except SS, significantly affected the villi surface area, antibody titer, and lymphocyte and heterophil percentages; however, the feedings were still not optimal for enhancing antibody titers and total and differentiated leukocytes. All types of selenium were demonstrated to increase the activity of GSH-Px significantly compared to the control treatment (p<0.05). Furthermore, the daily gain and feed conversion ratio of the groups treated with SY and HSM was significantly improved compared to that of the control group.

**Conclusion:**

*In ovo* SY and HSM improve immunity significantly, villi surface areas and performance. Therefore, both types are the best nutrient ingredients of IOF for building immunity and producing good performance in chickens.

## Introduction

In today’s farming industry, there is a continuing effort to enhance chicken productivity. Furthermore, many breeding programs are developing chickens with higher growth and feed efficiency. As a result, these chickens have a higher metabolic rate and are vulnerable to oxidative stress; the presence of free radicals may lower their performance. Thus, the antioxidant defense is needed for the chickens. Numerous factors may affect a chicken’s antioxidant state, causing oxidative stress in various phases of its development, such as before and after hatching [1]. The effort to anticipate and mitigate the risk of oxidative stress can be made through a nutritional approach such as the supplementation of selenium, which has a crucial role in various selenoproteins, particularly the activity of glutathione peroxidase (GSH-Px), in the body. GSH-Px is a cellular antioxidant capable of protecting cells from various damages by free radicals and the peroxidation and oxidation of polyunsaturated fatty acids [2].

Selenium is mainly given to the chickens using the conventional method, that is, through feed after the eggs have hatched. There were several attempts to supply selenium to the embryos as early as possible. Such a technique is known as *in ovo* feeding (IOF), during which nutrient fluid is injected into the egg amnion to allow the chicken embryos to consume the nutrient orally before hatching [3]. The selenium used in conventional or *in ovo* methods is organic, such as selenium yeast (SY) and selenomethionine, or inorganic, such as selenite and selenate. A chicken naturally consumes the amnion before hatches [4]; thus, injecting nutrients into the embryo’s amniotic fluid before it hatches will supply essential nutrients to the embryo’s intestine. Several experiments on IOF with selenium reported that *in ovo* selenium could improve the expression of immune genes mediated by broiler chicken cells [5], enhanced the immune and antioxidant response in the chickens exposed to the pathogens of necrotic enteritis when hatching [6], reduced oxidative damage during the incubation and neonatal periods [7], increased the adipose tissue mass, and caused adipocyte hypertrophy during the chicken embryo development [8], increased the villi length of the small intestine and the width of duodenal villi [9], and boosted the hatching weight of chicks, and augmented the final body weight, weight gain, and feed efficiency of the chickens [10].

The effectiveness of various types of selenium is still a subject of debate in the published literature. Thus, it is interesting to study the use of several types of selenium through IOF. This study also focused on the Kampung Unggul Balitbangtan (KUB) chickens, which were selected local chickens in Indonesia; the information on them was still scarce. The present study aimed to investigate the response of the local chickens to the IOF of three types of selenium, in terms of immunity, villi surface area, and early growth performance, to identify the best form of selenium as a nutrient ingredient of IOF.

## Materials and Methods

### Ethical approval

This study protocol was approved by The Institutional Animal Care and Use Committee, Indonesian Agency for Agricultural Research and Development (IAARD) (Approval Letter No. Balitbangtan/Balitnak/A/02/2019).

### Study period and location

The study was conducted from January to December 2019. All parameters were observed in the Laboratory of Indonesian Research Institute for Animal Production, except for the immunity parameters that were carried out at Indonesian Research Center for Veterinary Science.

### Materials

The fertile eggs used in the experiment were obtained from commercial breeding farms. Three types of selenium, organic SY, inorganic hydroxy-selenomethionine (HSM), and inorganic sodium selenite (SS), were purchased from feed additive distribution.

### Procedures of pre-hatch

In this study, 480 fertile KUB chicken eggs laid by local Indonesian chickens, with 120 eggs per treatment, were used in a single factor-completely randomized design with four IOF treatments. The four treatments included organic (SY; the T1 group), organic (HSM; T2), inorganic (SS; T3), and uninjected selenium (control; T4). The three types of selenium were dissolved in phosphate-buffered saline (PBS) and used at the concentration of 0.15 ppm [11].

All the IOF materials were dissolved in PBS and given to the eggs through a 0.5-ml injection per egg according to previous study [12]. However, the method was modified since the materials used in this study were local chicken eggs, which differed in size from the eggs by purebred chickens. The injections were performed at the egg amnion using a 20G needle (0.9 mm in diameter and 8 mm in length). The injections were done manually on day 18, when the incubated eggs were transferred from the setter to hatcher.

### Procedures of post-hatch

Once hatched, the chicks were placed in the same treatment as the previous egg injection treatment, namely four treatments with a completely randomized design. Each treatment only used 24 hatched chicks placed in three cages. The cages were placed in the room equipped with an automatic heater to control the temperature so that the lamps only functioned as lighting. The temperature was set at 32°C-35°C for the 1-7-day-old chicks, 29°C-31°C for the 8-21-day-old chicks, and 27°C-29°C for the 22-28-day-old chicks. Feed and drink were given *ad libitum* for 21 days. The formula diet for all the treatments was the same at the metabolizable energy of 2850 kcal/kg. The diet consisted of 63.14% of local corn, 32.29% of soybean meal, 0.38% palm oil (CPO), 1.69% limestone, 1.63% MDC phosphate, 0.46% salt, 0.25% premix, and 0.16% DL-methionine. The calculated nutrient content was 91.45% dry matter, 20.00% crude protein, 4.66% crude fiber, 3.03% crude fat, 7.48% ash, 1.00% calcium, and 0.45% phosphor, 1.055% lysine, and 0.477% methionine. The total of selenium in the feed was approximately 0.13 ppm. On 4th day, the chicks were vaccinated with Newcastle Disease vaccine (live) by eye drop.

### Observed variables

The chicks’ growth performance metrics, including body weight, weight gain, feed consumption, and feed conversion ratio, were recorded on post-hatch days 0-21. On day 7, villi samples were taken from one chicken that was slaughtered in each experimental replicate to measure the villi surface area according to the method used by Iji *et al*. [13]. Blood samples were taken from five chickens in each replicate on days 4, 11, and 18. The antibody titers on days 4, 11, and 18 were analyzed using the hemagglutination inhibition (HI) test method. The total number and differentiation of leukocytes on day 18 were counted according to the method of Benjamin [14]. GSH-Px enzyme activity on day 18 according to the method used by Kotan *et al*. [15]. All the data were analyzed with analysis of variance using SAS software version 9.1 (SAS Inc., NC, USA).

## Results

### HI-test

The antibody titer for the treatment groups ranged from 3.3 to 3.6 Log2 (Table-1). The titers increased 1 week after vaccination to 7.3 Log2 for the T1 and T2 groups and 5.0 Log2 for T3 and T4, then decreased 2 weeks after vaccination to 5.0 Log2 (T1/T2), 4.6 Log2 (T3), and 4.0 Log2 (T4). The effect of vaccination on antibody titers was significantly noticeable (p<0.05) after 1 and 2 weeks of vaccination.

**Table-1:** KUB chicken’s response to *in ovo* Se injection on antibody titer, total, and differentiation of leukocyte, villi surface area, and GSH-Px enzyme activity.

Parameter	T1 (SY)	T2 (HSM)	T3 (SS)	T4 (Control)	SEM	p-value
Antibody titer (Log 2)						
4 days old (day of ND vaccination)	3.3	3.3	3.6	3.3	0.31	0.2575
11 days old (7^th^ day post-vaccination)	7.3^a^	7.3^a^	5.0^b^	5.0^ b^	0.42	0.0072
18 days old (14^th^ day post-vaccination)	5.0^a^	5.0^a^	4.6^ab^	4.0^ b^	0.3	0.006
Total Leukocyte (x10^3^/mm^3^) and differentiation of leukocyte (%)						
Total of leukocyte	28.50	29.16	27.10	25.47	0.39	0.0533
Lymphocyte	53.00^a^	55.00^a^	45.00^ab^	39.00^b^	1.79	0.0143
Heterophile	35.67^a^	34.66^a^	31.33^b^	30.66^b^	0.64	0.0115
H/L ratio	0.68	0.63	0.70	0.78	0.02	0.4185
Eosinophil	9.66	9.33	7.66	5.66	0.56	0.1228
Basophil	2.33	2.00	2.00	1.67	0.22	0.9643
Monocyte	3.66	2.00	1.66	1.33	0.27	0.0779
GSH-Px enzyme (U/L)	1124.1^a^	1118.8^a^	1016.8^a^	616.2^b^	53.19	0.0267
Villi surface area (mm^2^)						
Duodenum	0.019^a^	0.017^a^	0.014^a^	0.008^b^	0.71	<0.0001
Jejunum	0.019^a^	0.020^a^	0.013^b^	0.013^b^	0.62	0.0016
Ileum	0.042^a^	0.038^a^	0.034^a^	0.024^b^	1.28	<0.0001

Means in the same row with different superscripts differ significantly (p<0.05). SY=Selenium yeast, HSM=Hydroxy-selenomethionine, SS=Sodium selenite, GSH-Px=Glutathione peroxidase enzyme, SEM=Standard error of means. Se=Selenium, H/L=Heterophile-to-lymphocyte

### Total and differentiation of leukocyte

The total number of leukocytes in the treatment groups ranged from 25.47 to 29.19×10^3^/mm^3^ (Table-1). The T2 group had the highest number of leukocytes, and T4 had the lowest, but the difference in these values was not statistically significant (p>0.05). The lymphocyte count in T1 at 53.00×10^3^/mm^3^ and T2 at 55.00×10^3^/mm^3^ was significantly higher than that in T4 at 39.00×10^3^/mm^3^ (p<0.05). Similarly, the heterophile count in T1 and T2, at 35.67×10^3^/mm^3^ and 34.66×10^3^/mm^3^, respectively, was higher than that in T4 at 30.66×10^3^/mm^3^. The heterophile-to-lymphocyte (H/L) ratios ranged from 0.63 to 0.78. The numbers of other differentiated leukocytes displayed no significant difference, with 5.66–9.66×10^3^/mm^3^ for eosinophils, 1.67–2.33×10^3^/mm^3^ for basophils, and 1.33–3.66×10^3^/mm^3^ for monocytes (p>0.05).

### Activity of GSH-Px

The activity of GSH-Px in the *in ovo* selenium treatment groups (Table-1) exhibited a significant difference between the T1, T2, and T3 groups, and the T4 control group (p<0.05). The GSH-Px activity in T1, T2, and T3 increased drastically to 1124.1, 1118.8, and 1016.8 U/L, respectively, compared to the control at 616.2 U/L.

### Villi surface area

**Figure-1:**
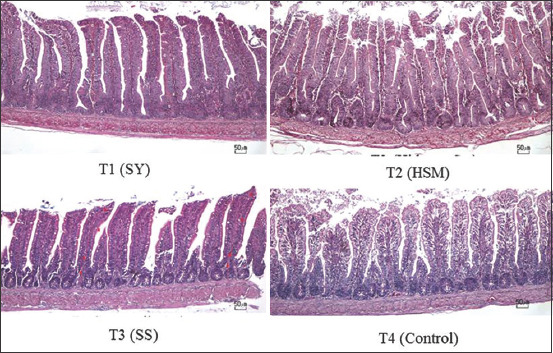
Villi display in illeum from T1 (selenium yeast), T2 (hydroxy-selenomethionine), T3 (sodium selenite), T4 (Control).

The villi of the ileum of the chicks in all treatments (Figure-1) resembled the normal villi. There were indications that the villi surface area at the end of the villi channel was increased in selenium treatments. The chickens with *in ovo* selenium treatment had significantly larger villi surface areas, at 0.019 mm^2^ for T1, 0.017 mm^2^ for T2, and 0.014 mm^2^ for T3, in the duodenum than that of the T4 control group at 0.008 mm^2^. The ileum’s villi surface area was also larger in the *in ovo* selenium-treated groups, at 0.042 mm^2^ for T1, 0.038 mm^2^ for T2, and 0.034 mm^2^ for T3, than that of the T4 control group at 0.024 mm^2^. However, the surface areas in the jejunum were slightly different, where the i*n ovo* inorganic Se treatment (T3) group had the same villi surface area as the control (T4) group, but significantly (p<0.05) smaller than the *in ovo* SY (T1) and *in ovo* HSM (T2) treatment groups.

### Early growth performance of the KUB chickens

The performance of the chicks was observed for 21 days post-hatch after they were treated with *in ovo* selenium on the 18^th^ day of incubation (Table-2). The body weight ranges from 47.12-60.44 g at week 1, 82.55-87.94 g at week 2, and 136.22-145.33 g at week 3. The average body weight of T1 and T2 at each week was significantly higher than that of T4. This trend was in line with the increase in daily weight gain, ranging from 18.83 to 25.88 g for the *in ovo* selenium treatment (T1, T2, and T3) groups and 13.09 g for the control treatment (T4) group at week 1. In contrast, the range was 51.78-53.84 g for the *in ovo* selenium treatment groups and 48.54 g for the control group at week 2. Body weight gain at week 3 was not significant (p>0.05) for the treatment groups, ranging from 102.2 to 111.56 g.

**Table-2:** Chicken’s response to *in ovo* Se injection regarding average daily weight increase, feed consumption and conversion.

Parameter/age	T1 (SY)	T2 (HSM)	T3 (SS)	T4 (Control)	SEM	p-value
Daily body weight (g/bird)						
7 days	59.65^a^	60.44^a^	52.25^ab^	47.12^b^	1.37	0.0020
14 days	87.60^a^	87.94^a^	85.10^ab^	82.55^b^	0.73	0.0185
21 days	145.33^a^	144.46^a^	141.69^ab^	136.22^b^	1.06	0.0416
Daily weight gain (g/bird)						
7 days	25.88^a^	26.05^a^	18.83^b^	13.09^c^	1.35	0.0033
14 days	53.84^a^	53.55^a^	51.78^ab^	48.54^b^	0.71	0.0173
21 days	111.56	110.07	108.27	102.2	1.09	0.0635
Daily feed consumption (g/bird)						
7 days	77.91^a^	77.64^a^	55.13^b^	42.54^c^	3.76	0.0056
14 days	165.89	165.09	162.83	154.29	1.83	0.1985
21 days	347.72	343.29	347.85	337.44	2.86	0.8963
Feed conversion						
7 days	3.01^a^	2.98^a^	3.14^b^	3.25^c^	0.03	0.0033
14 days	3.08^a^	3.08^a^	3.14^ab^	3.18^b^	0.01	0.0193
21 days	3.11^a^	3.12^a^	3.21^b^	3.30^c^	0.02	0.0012

Means in the same row with different superscripts differ significantly (p<0.05). SY=Selenium yeast, HSM=Hydroxy-selenomethionine, SS=Sodium selenite, SEM=Standard error of means. Se: Selenium

On the other hand, selenium treatment affected the daily feed consumption only in the 1^st^ week (p<0.05). The daily feed consumption values were 42.54-77.91 g at week 1, 154.29-165.89 g at week 2, and 337.44-347.72 g at week 3. The feed conversion ratio was significantly (p<0.05) affected by the *in ovo* selenium treatment. At 21 days, T1 and T2 had d better feed conversion ratios than T3 and T4.

## Discussion

In the present study, different types of selenium did not significantly affect the hatchability (87.5–90%) and hatching weight (33.15–34.39 g) of KUB local chicken. Similarly, no effect of *in ovo* selenium on hatchability and hatching weight was reported on the broilers and other breeds of chicken [10,12,16].

The information regarding the effect of certain materials on a chicken’s immunity is important, considering the duration of its maternal immunity is relatively short [17]. One of the methods to obtain such information is by observing measuring the antibody titer. The use of vaccines may provide indicators on the antibody titer since vaccines contain antigens that stimulate antibody formation [18]. In general, the antibody titer in this study increased 1 week after vaccination and then decreased after 2 weeks (Table-1). It was assumed that the increase in antibody titer occurred due to live ND vaccination, which typically caused a rapid effect on antibody formation. On the other hand, inactivated ND vaccine induced a slower response due to the adjuvants in the form of an emulsion, which resulted in slower antigen release [19].

Almost all types of selenium, except inorganic selenium (T3), exhibited a significantly positive effect on the antibody titer 7 and 14 days after vaccination compared to the control group (p<0.05). A higher antibody titer indicates an enhanced immune response, as reported by other researchers [5], who found that *in ovo* selenium resulted in a better immune response and an increase in the expression of immune genes.

This experiment found that IOF with SY enhanced the early growing chicken’s immunity more than inorganic selenium. SY could be absorbed efficiently since it contains the functional component of the body’s selenoprotein and amino acids; besides, its absorption was higher than inorganic selenium [20]. Hematological assessment toward the number of total and differentiated leukocytes may indicate a health and immune status of the animal [21]. Here, the average total leukocytes ranged from 25.47 to 29.16×10^3^/mm^3^ (Table-1). The total leukocyte count (p<0.05) during the early growing period was increased by the *in ovo* selenium injection of SY (T1) and HSM (T2) compared to inorganic selenium (T3) and control (T4). A similar result was reported by other researchers [10] that *in ovo* selenium injection slightly increased total leukocyte count in 7-day-old broiler chicks; however, the increase was not significant.

The effect of selenium treatment on leukocyte differentiation varied (Table-1). The lymphocyte and heterophile counts were significantly affected, while monocytes, eosinophils and basophils were not affected by the selenium treatments. All the selenium treatments resulted in a positive effect on the lymphocyte count compared to the control group. These results indicated that *in ovo* selenium injection had a rapid and positive effect on antibody formation and cellular immunity development. Without selenium acting as a glutathione component, the lymphocyte cannot produce antibodies against infection and enhance the body’s immune system [2].

Another type of differentiated leukocytes significantly affected by selenium supplements was heterophile. The heterophile count in the T1 and T2 groups exhibited a significant difference compared to the control group (p<0.05); only the heterophils in the T3 (inorganic Se) group did not exhibit a significant difference compared to the control (p>0.05). It is assumed that SY contains glucan from the *Saccharomyces cerevisiae* cell wall as an active component that plays a crucial role in the non-specific stimulation of immune response [22]. Therefore, using organic selenium, a combination of selenium and yeast may affect the immune response. SY and HSM play a positive role in the formulation of heterophils, which are phagocytic and act as the front-line defense against disease resulting from infection or inflammation. The ratio of H/L ratio is often used to indicate the stress condition of animals; the ratio typically is positively correlated with heat stress [23]. The H/L ratio in the present study was not significantly different among the treatment groups, and its score was below 0.8 (ranged from 0.62 to 0.78), and the animals were thus categorized to be under a moderate level of stress [24].

Selenium serves as an essential mineral in antioxidant defense and is a pivotal component of GSH-Px; it is critical to the effective synthesis of GSH-Px [25]. GSH-Px plays a key role in cellular antioxidant defense and reduces free radicals, such as H_2_O_2_ or other hydroperoxides, into water, or alcohol bonds [2]. Here, all types of *in ovo* selenium treatment yielded a significant effect on GSH-Px activity compared to the control (p<0.05) (Table-1). The increase of GSH-Px was also reported by others who administered selenium to chickens [26,27]. GSH-Px activity is substantially affected by the availability of selenium. Thus, supplying 0.15 ppm of selenium through IOF is considered sufficient to protect the embryo development from tissue damage by free radicals and simultaneously enhance the egg’s survivability until it hatches. Thus, selenium’s critical benefit is associated with its ability to protect the embryo development from peroxidation during embryogenesis [27].

The results of the study indicate that three types of *in ovo* selenium injection can be used to overcome the emergence of free radicals because of the stress caused by the rapidly increasing metabolic rate and oxygen consumption during the neonatal period, the possible heat stress effect, or other environmental threat.

Digestive ability and food nutrient absorption may be affected by the intestinal epithelial surface area and villi surface area in the duodenum, jejunum, and ileum [28]. The villi in the ileum from the chicks in each treatment group (Figure-1) display the characteristics of the normal villi. It is difficult to determine the best treatment visually since observations would be descriptive and highly subjective. However, it can still provide a comparative depiction quickly. Calculating the villi surface area can be a quantitative approach in determining the animal’s responses to a selenium treatment.

All types of selenium had a positive effect on the villi surface area compared to the control treatment (Table-1). The scores were significantly different in the treatment groups compared to the control (p<0.05), except for T3 in the jejunum, likely due to SS, as inorganic selenium, undergoing structural change into ions. Because of the acidic condition in the proventriculus, selenite is less efficient in stimulating villi development. In contrast, SY and HSM are expected to be more tolerant toward acid conditions. SY (organic) contains β (1-3) glucan***s*** and β (1-6) glucans, which are soluble in phosphate-buffered saline [29]. Glucan can be bind macrophages and natural killer cells that activate the macrophages [30], thus preventing pathogen growth and allowing intestinal villi to develop. In addition, SY contains 90% selenomethionine [31]. Both SY and HSM are easily absorbed into all intestinal segments. Meanwhile, inorganic selenium, such as selenite, is only absorbed efficiently in the ileum [32]. Different levels of selenium absorption in different parts of the intestine can lead to differential intestinal development.

Different responses to villi area development due to selenium treatment may be associated with different mucin production. Mucin is the primary component of the mucous membrane involved in the filtration, digestion, and absorption of nutrients in a digestive tract [33]. There is a positive correlation between the villi surface area and the mRNA level of the mucin gene [34]. Therefore, giving selenium as early as possible through IOF likely helps accelerate intestinal development, eventually benefiting the digestive process and nutrition absorption. SY and HSM can improve villi development.

The early growing period is typically a crucial phase that can be used as a success indicator of IOF technology. The present study showed that the selenium-treated chicks exhibited a higher average of body weight at each week than the control chicks, except the T3 chicks, which showed a statistically insignificant difference compared to the control treatment. The average daily weight gain of the chicks on days 0-7 and 0-14 was affected significantly by selenium treatments (p<0.05). However, selenium’s effect was insignificant when the observation was done on days 0-21, despite the tendency of difference between the selenium and control treatment groups (p>0.05). The first 2 weeks of the growing period are crucial because the young chicks are exposed to many diseases; therefore, high immunity defense that was obtained by selenium treatment could prevent those diseases, hence improved the performance.

IOF with selenium results in better feed efficiency in the early growing chickens. Feed intake was similar for all treatments on days 0-21, but *in ovo* selenium treatments resulted in better feed conversion than the control treatment. Better feed conversion ratio is associated with better villi surface area, as shown in Table-1. Rapid intestine villi development is beneficial for digestion and nutrition assimilation, thus increasing body weight [35]. The better formulation of antibody titer, differentiation of leukocyte, and higher GSH-Px activity are likely factors supporting the growth of chickens treated with *in ovo* selenium injection. It was reported that IOF with selenium for broilers increased intestine development, body weight, growth, and feed conversion, resulting in better early growth [10].

## Conclusion

Except for inorganic selenium, *in ovo* selenium injection significantly affected villi surface area, antibody titer, and lymphocyte and heterophil ratios. However, all types of selenium were shown to increase the activity of GSH-Px. The daily gain and feed conversion ratio at 21 days was improved significantly by IOF of SY and HSM. Therefore, SY and HSM can be recommended as the best nutrient ingredient of IOF for building immunity and producing good growth performance in chickens.

## Authors’ Contributions

RK designed and performed the experiment, collected the data, and wrote the manuscript. YR, RM, AJ designed, and supervised the experiment, checked the data analysis, and revised the manuscript. BT supervised data analysis and table presentation. EW supervised the experiment and revised the method and discussion of the manuscript. All authors read and approved the final manuscript.
